# Association of ABO blood types with ST resolution following thrombolysis in acute ST elevation myocardial infarction

**DOI:** 10.34172/jcvtr.2020.18

**Published:** 2020-05-26

**Authors:** Ahmad Separham, Soudabeh Dinparvar, Safa Savadi-Oskouei, Leili Pourafkari, Aidin Baghbani-Oskouei, Nader D Nader

**Affiliations:** ^1^Cardiovascular Research Center, Tabriz University of Medical Sciences, Tabriz, Iran; ^2^Shahid Beheshti University of Medical Sciences, Tehran, Iran; ^3^Department of Anesthesiology, University at Buffalo, Buffalo, New York, USA

**Keywords:** ABO Blood Group, Acute Myocardial Infarction, ST-Segment Resolution, Thrombus Burden, Thrombolysis

## Abstract

***Introduction:*** There is paucity of data about the possible role of ABO antigen in response to pharmacologic reperfusion therapy in ST-segment elevation myocardial infarction (STEMI) and its relationship with ST segment recovery; thus, we aimed to evaluate the association of ABO antigen with ST-segment resolution in STEMI patients treated with thrombolysis.

***Methods:*** This prospective and observational study was conducted between March 2016 and September 2017 on patients with first acute STEMI within the first 12 hours after onset of symptoms treated with thrombolysis. Myocardial reperfusion success was determined by single-lead ST-segment recovery in 12-lead ECG. Patients were considered as responders if ST-segment resolved ≥50% or were assigned as non-responders if ST-segment resolution was <50%. Univariable and multivariable analyses were performed to examine the contribution of "A" and "B" blood group antigens to ST-segment resolution and the occurrence of major adverse cardiovascular or cerebrovascular event (MACCE). Odds ratio (OR) with 95% confidence interval (CI) were reported for each variable.

***Results:*** In this study 303 patients (187 males and 116 females) with a mean age of 56.6 ± 16.8 (ranging from 39 to 87 years) were enrolled. 184 patients (60.7%) were responders and 119 patients (39.2%) were non-responders. The presence of either A (4.5 folds increase) or B (5.4 folds increase) antigen was associated with a higher likelihood of a response to thrombolytic therapy, while had not effect on the occurrence of MACCE.

***Conclusion:*** We conclude that the presence of A or B blood group antigens is associated with a better response to thrombolytic therapy in patients with acute STEMI. This finding may imply a higher likelihood for thrombotic occlusion of coronary arteries in patients who have either A or B antigen in their blood.

## Introduction

Ischemic heart disease (IHD) is the most important cause of morbidity and mortality in developed countries.^1^ Acute ST-segment elevation myocardial infarction (STEMI) is generally associated with a significant injury to the myocardium, and no treatment or late treatment portends poor prognosis.^2^ The myocardial blood flow establishment is accomplished through reperfusion therapy by either thrombolysis or primary percutaneous coronary intervention (PCI), which results in ST-segment resolution in the electrocardiogram (ECG) of STEMI patients.^3^ Thrombolysis has been considered as the choice treatment for STEMI when PCI cannot be performed timely.

Nowadays, ABO blood group is suggested to be a risk factor for the development of several vascular diseases, such as hypertension,^4^ thromboembolism,^5^ and coronary artery disease (CAD).^6-8^ The ABO gene locus has been mapped to chromosome 9 at locus 9q34^9^ and blood group antigens (A, B, and H) have been demonstrated to consist of complex carbohydrate molecules, which are placed on the extracellular surface of the red blood cell (RBC) membrane; however, they are actually expressed on a variety of human tissues i.e. epithelium, sensory neurons, platelets, and vascular endothelium.^10^ Additionally, it seems that ABO blood group is associated with serum lipids’ metabolism,^11^ and there is a great interest about the impact of ABO blood groups on cardiovascular risk factors.

There is a paucity of data about the possible role of ABO antigen in response to pharmacologic reperfusion therapy in STEMI and its relationship with ST segment recovery; thus, we aimed to evaluate the association of ABO antigen with ST-segment resolution in STEMI patients treated with thrombolysis. More specifically, we hypothesized that the presence of A or B antigens modify the therapeutic effect of thrombolysis through their effects on coagulation.

## Materials and Methods

### Study design and settings

This study included a prospective cross-sectional comparison of ST-segment resolution response in patients who were admitted to a university-affiliated heart center with evidence of acute STEMI between March 2016 and September 2017 and received thrombolysis. The study design, protocols, and informed consent forms were reviewed and approved by the institutional review board for its merit and ethics in human subject research. All patients signed a partial waiver form for Health Insurance Portability and Accountability Act to allow the investigating team to screen their medical records for study eligibility. Following the screening process, all patients who met the inclusion criteria were asked to sign consent for the study prior to initiation of thrombolysis.

### Participants

All admitted patients to the medical center with first acute STEMI, who were experiencing symptoms no longer than 12 hours were screened for possible inclusion. Patients deemed eligible for primary PCI underwent primary PCI and were not included in this study. Those who could not undergo primary PCI and for various reasons mainly unavailability of catheterization lab, and received thrombolysis at the discretion of consultant cardiologist were screened for inclusion. Patients with any contraindication to the use of thrombolytic medications, such as aortic dissection, systolic blood pressure (SBP) >180 mm Hg, history of head trauma or any surgical procedure within past 90 days, and history of cerebrovascular accident within the past 90 days did not receive thrombolysis and were not included. Patients who had known hypersensitivity reaction to reteplase, previous history of myocardial infarction, left bundle branch block (LBBB) pattern in ECG, cardiogenic shock at admission, and patients who refused to participate were also excluded. Electrocardiographic measurements were performed first on presentation, immediately before thrombolytic therapy, and then, 90 minutes after fibrinolysis. All patients received acetyl salicylic acid (325 mg), clopidogrel (300 mg in patients younger than 75 and 75 mg in patients above 75 years old), unfractionated or low molecular weight heparin, and statins on admission in the emergency department. All qualifying patients received the standard thrombolysis regimen at the institute consisting of two intravenous bolus doses of 10 units of reteplase (OSVE Pharmaceutical Co. Tehran, Iran) administered 30 minutes apart.

### Outcome and independent variables

Patient clinical and demographic characteristics including age, smoking history, medication use and history of hypertension, diabetes mellitus, hyperlipidemia, chronic kidney disease, cerebrovascular disease, as well as family history of CADs, systolic and diastolic blood pressures at admission were recorded. Peripheral blood samples for each participant were collected in citrate tubes at admission time, which were stored until they were analyzed within 24-hours of collection. Biochemical measurements including serial and peak creatine kinase (CK) with MB fraction, cardiac troponin-I, serum glucose and creatinine, and full lipid profile including total cholesterol, high-density lipoproteins, low-density lipoproteins and triglycerides concentrations were performed. Blood typing for ABO and Rh antigen and complete blood cell count were collected from the patient clinical records. Blood group antigen was the main predictor variable of interest in this study which was test against all potential confounders, as above.

STEMI was defined according to the criteria published by the American College of Cardiology as clinical symptoms suggestive of acute myocardial ischemia with increased cardiac biomarkers above the 99th percentile upper reference limit accompanied by new ST-elevation defined by ≥1 mm at the J-point in two contiguous leads in all leads other than leads V2–V3 and ≥2 mm in men ≥40 years; ≥2.5 mm in men <40 years, or ≥1.5 mm in women in leads V2-V3.^12^ The magnitude of ST segment elevations were assessed in all involved leads (V1 through V6, I, aVL for the anterior myocardial wall, and II, III and aVF for the inferior wall). Myocardial reperfusion was the primary outcome variable of this study as determined by ≥50% resolution in single-lead ST-segment elevation within 90 minutes after thrombolysis when it was compared to its baseline value. Patients were considered as responders if ST-segment resolved ≥% 50, otherwise were considered as non-responders.^13^ The occurrence of a composite major adverse cardiovascular and cerebrovascular events (MACCE) was considered a secondary outcome variable to this study. This variable refereed to any single or combined occurrence of myocardial re-infarction, stroke, acute heart failure (cardiogenic shock/pulmonary edema), and/or death.

Family history of atherosclerotic cardiovascular disease in at least one first-degree male relative before 55 years or before 65 years of age in a first-degree female relative was considered as a family history of premature cardiovascular disease. Hypertension was defined as either SBP of ≥140 mm Hg, diastolic blood pressure (DBP) ≥90 mm Hg (measured at least two times) or current use of any anti-hypertensive medications in combination with a self-report of hypertension. The diagnosis of diabetes was defined as meeting at least one of the following criteria: fasting blood glucose (FBG) ≥126 mg/dL, 2-hour post prandial glucose (2h-PPG) ≥200 mg/dL, or taking anti-diabetic medications. Hyperlipidemia was defined as total cholesterol of >200 mg/dL or taking lipid-lowering agents.

### Sample size determination

Power analysis and sample size determination was performed using G*Power statistical tool and online calculator provided to the public by the website of the University of British Columbia. The frequency of ST-segment resolution (≥50%), the primary outcome variable, was used for calculating the sample size. In our previous work, significant ST-segment resolution occurred in 57.3% of the STEMI patients receiving thrombolysis.^14,15^ A 20% difference in the prevalence was clinically considered as significant. A total of 97 patients were required in each group to detect this difference. Additionally, based on our previous work using a patient population from similar demographic and geographic distribution, the prevalence of patients with either A and/or B blood group antigens were 68% while those patients lacking the either antigen (O Blood group) was 32%.^16^ Therefore, a total of 303 patients were enrolled to meet the minimum of 97 patients with O blood group. With a sample of this size the power of the study was 0.80 with 95% confidence interval.

### Quantitative variables and statistical methods

Data were expressed as value and percentage for categorical variables. All numeric variables were tested using Kolmogorov–Smirnov test for the presence of a normal distribution and mean and standard deviation (SD) were used to express numerical variables with a normal distribution. Since all continuous variables had normal distribution, they were tested with independent t-test. Categorical data were also analyzed using chi-square test and Fisher exact test, as appropriate. Pearson analyses were used to correlate ABO blood group antigens and other clinical variables with favorable response to thrombolysis (ST segment resolution) and incident MACCE as the dependent outcome variables. Odds ratio (OR) with 95% confidence interval (CI) were reported for each variable, both after univariate and multivariate analysis. *P* values less than 0.05 were considered statistically significant. All analyses were performed using SPSS version 25.0 (IBM Inc. Chicago, IL).

## Results

A total of 352 patients were screened; from which, 315 fulfilled the inclusion criteria. After excluding patients with incomplete laboratory data, 303 patients (187 males and 116 females) with a mean age of 56.6 ± 16.8 (ranging from 39 to 87 years) were enrolled in this study.

Table 1 illustrates clinical characteristics as well as the laboratory findings at the time of admission in subgroups of non-responders (ST resolution <50%) and responders (ST resolution ≥50%); 184 patients (60.7%) were responders and 119 patients (39.2%) were non-responders. The non-responder group had higher glucose level and longer ischemic time, when compared to the responders. Furthermore, the presence of A or B antigens was significantly higher in the responders group. However, there was no significant difference in other characteristics of these two groups.

**Table 1:** Cross-tabulation and univariate analysis of characteristics of patients based on their resolution response to fibrinolytic treatment following ST elevation myocardial infarction (STEMI)

	**ST Resolution <50%**		**ST Resolution ≥50%**		***P*** **value**	**OR**	**95% Confidence Interval**	
	**(n=119)**		**(n=184)**					
Family history	3	2.5%	4	2.2%	1.000	0.859	0.189	3.909
Hyperlipidemia	16	13.4%	18	9.8%	0.354	0.698	0.341	1.430
Hypertension	51	42.9%	61	33.2%	0.091	0.661	0.411	1.064
Diabetes mellitus	36	30.3%	33	17.9%	0.017	0.504	0.293	0.867
Prior or current smoking	44	37.0%	84	45.7%	0.154	1.432	0.893	2.295
Chronic kidney disease	6	5.0%	3	1.6%	0.162	0.312	0.077	1.273
Cerebrovascular Diseases	1	0.8%	0	0.0%	0.393	0.391	0.339	0.450
Prior PCI	4	3.4%	2	1.1%	0.216	0.316	0.057	1.753
Prior CABG	1	0.3%	0	0.3%	0.393	0.391	0.339	0.450
Prior myocardial Infarction	5	4.2%	8	4.3%	1.000	1.036	0.331	3.247
Chronic stable angina	3	2.5%	3	1.6%	0.683	0.641	0.127	3.229
A agglutinogen	37	31.1%	93	50.5%	0.001	2.265	1.396	3.675
B agglutinogen	28	23.5%	85	46.2%	<0.001	2.790	1.670	4.662
A or B antigens	62	52.1%	153	83.2%	<0.001	4.537	2.677	7.690
Statin drugs	10	8.4%	10	5.4%	0.348	0.626	0.253	1.554
Diuretics	4	3.4%	5	2.7%	0.742	0.803	0.211	3.053
Acetyl salicylic acid	11	9.2%	12	6.5%	0.384	0.685	0.292	1.607
ACE Inhibitors/ARBs	17	14.3%	21	11.4%	0.481	0.773	0.389	1.534
Calcium channel blockers	4	3.4%	5	2.7%	0.742	0.803	0.211	3.053
Digoxin	1	0.3%	0	0.3%	0.393	0.391	0.339	0.450
Clopidogrel	2	1.7%	5	2.7%	0.708	1.634	0.312	8.562
Beta blockers	16	13.4%	20	10.9%	0.586	0.785	0.389	1.584

Note: The state of resolution was defined by more than or equal to 50% reduction in the sum of ST elevation voltage from its baseline value.

Table 2 summarizes the characteristics of the study population by ABO blood groups. Patient characteristics including coronary risk factors, medical co-morbidities and medications were similar between groups. Laboratory findings and clinical outcome of patients are also shown in Table 3 according to the presence of A and/or B antigens.

**Table 2:** Patient characteristics according to their blood group antigens

	**“A” Antigen (+) (n=130)**		**“B” Antigen (+) (n=113)**		**Antigen Negative (n=88)**		***P*** **value**
	**No.**	**%**	**No.**	**%**	**No.**	**%**	
Family history of coronary disease	5	3.8%	3	2.7%	1	1.1%	0.715
Hyperlipidemia	14	10.8%	9	8.0%	13	14.8%	0.231
Hypertension	45	34.6%	46	40.7%	34	38.6%	0.697
Diabetes Mellitus	28	21.5%	35	31.0%	14	15.9%	0.072
Current or prior smoking	59	45.4%	40	35.4%	40	45.5%	0.522
Chronic kidney diseases	3	2.3%	3	2.7%	4	4.5%	0.778
Prior coronary revascularization	3	2.3%	2	1.8%	3	3.4%	0.297
Prior myocardial infarction	6	4.6%	4	3.5%	3	3.4%	0.763
Chronic stable angina	2	1.5%	2	1.8%	3	3.4%	0.093
Statin drugs	10	7.7%	6	5.3%	6	6.8%	0.854
Antiplatelets: ASA or clopidogrel	12	9.2%	7	6.2%	6	6.8%	0.388
ACE Inhibitors/ARBs	16	12.3%	16	14.2%	9	10.2%	0.977
Calcium channel blockers	2	1.5%	3	2.7%	4	4.5%	0.290
Beta blockers	12	9.2%	12	10.6%	14	15.9%	0.073
Anterior myocardial infarction	81	63.8%	59	52.2%	54	61.4%	0.056
Inferior myocardial infarction	45	35.4%	52	46.0%	31	35.2%	0.056
Lateral myocardial infarction	1	0.8%	2	1.8%	3	3.4%	0.056

**Table 3:** Laboratory values of the patients according to the presence of blood group antigens A or B agglutinogens

	**Status**	**“A” Antigen (n = 130)**		**“B” Antigen (n = 118)**		**Antigen Negative (n = 88)**	
		**Mean**	**SD**	**Mean**	**SD**	**Mean**	**SD**
Hemoglobin	No	14.6	7.9	14.8	7.5	14.3	2.3
	Yes	14.5	2.1	14.2	2.4	15.2	10.7
Platelets	No	219	69	219	66	223	72
	Yes	222	68	222	72	214	60
Neutrophil count (cells/nL)	No	7.4	3.2	8.0	5.1	8.3	5.0
	Yes	8.6*	5.8	7.8	3.2	7.1*	3.1
Lymphocyte count (cells/nL)	No	2.2	1.3	2.1	1.5	2.2	1.5
	Yes	2.1	1.6	2.2	1.3	2.1	1.1
Neutrophil/lymphocyte ratio	No	4.68	3.56	5.62	5.45	5.65	5.21
	Yes	6.26*	6.05	4.91	3.60	4.67	3.81
Platelet/lymphocyte ratio	No	129	87	146	117	143	108
	Yes	153	122	128	76	132	96
Serum creatinine (mg/dL)	No	1.21	0.52	1.22	0.63	1.17	0.45
	Yes	1.18	0.53	1.15	0.28	1.25	0.68
Glucose (mg/dL)	No	156	91	148	75	159	88
	Yes	154	76	167	97	144	75
Total serum cholesterol (mg/dL)	No	184	39	187	45	187	42
	Yes	189	46	185	38	186	42
Serum triglyceride	No	137	70	138	77	134	71
	Yes	137	78	135	68	143	79
High density lipoproteins (mg/dL)	No	39.4	16.9	40.2	15.1	39.5	10.8
	Yes	40.0	8.8	38.8	12.0	40.0	20.1
Low density lipoproteins (mg/dL)	No	107	28	108	36	110	33
	Yes	113	38	111	27	107	31

Asterisks show statistically significance.

Logistic regression analyses based on ST resolution of equal or greater than 50% and MACCE as the dependent variables are shown in Table 4. Serum glucose level and O blood group reduced the likelihood of a response to thrombolytic therapy; corresponding ORs (95% CI) were 0.993 (0.989-0.998) and 0.180 (0.101-0.319), respectively. Moreover, there was a trend for total ischemic time for reducing the favorable ST-segment recovery (OR: 0.998, 95% CI: 0.996-1.000, *P* = 0.092). Diabetes mellitus was found to be the only independent predictor of MACCE in our study population [OR: 3.262 (1.502-7.083)]. On the contrary, antiplatelet use, neutrophil/lymphocyte ratio (NLR), A and B antigens did not independently predict MACCE. Neither presence of A nor B antigen associated with the occurrence of MACCE in multivariate analysis (Table 5).

**Table 4:** Clinical outcome of the patients after ST elevation myocardial infarction according to their blood group antigens

	**Status**	**“A” Antigen (n = 130)**		**“B” Antigen (n = 118)**		**Antigen Negative (n = 88)**	
		**No.**	**%**	**No.**	**%**	**No.**	**%**
ST elevation resolution ≥ 50%	(--)	91	52.6%	99	52.1%	153	71.2%
	(+)	93*	*71.5%	85*	*75.2%	*31	*35.2%
Major advanced cardiac cerebral events (MACCE)	(--)	21	12.1%	24	12.6%	23	10.7%
	(+)	14	10.8%	11	9.7%	12	13.6%
Pump Failure	(--)	13	7.5%	15	7.9%	16	7.4%
	(+)	10	7.7%	8	7.1%	7	8.0%
Death within the Hospital	(--)	10	5.8%	9	4.7%	11	5.1%
	(+)	6	4.6%	7	6.2%	5	5.7%
Bleeding complications	(--)	9	5.2%	8	4.2%	11	5.1%
	(+)	5	3.8%	6	5.3%	3	3.4%
**Continuous Variables**	**Status**	**Mean**	**STD**	**Mean**	**STD**	**Mean**	**STD**
Left ventricular ejection fraction	(--)	39.4	8.0	38.3	7.8	38.6	8.0
	(+)	38.0	7.8	39.6	8.1	39.3	7.8
Maximum cardiac troponin I (ng/L)	(--)	14.8	11.5	16.0	11.4	15.2	11.3
	(+)	15.9	11.3	14.1	11.3	15.4	11.7
Maximum creatine kinase MB (I.U)	(--)	225	191	249	224	233	199
	(+)	247	216	210	156	238	211
Mean arterial pressure (mm Hg)	No	98	16	98	15	98	16
	Yes	98	15	99	17	98	15
Duration of hospital stay (days)	No	5.9	3.1	6.1	3.6	5.9	3.0
	Yes	6.0	3.4	5.5	2.3	6.0	3.6
Total Ischemia Time (min)	No	207	142	203	130	209	134
	Yes	208	125	214	143	204	138

**Table 5:** Logistic regression model for ST resolution (upper panel) and major adverse cardiac and cerebral events (MACCE) following thrombolysis for acute ST elevation myocardial infarction

**Outcome Variable: STR**	**Coefficients**	**SE**	***P*** **value**	**Odds Ratio**	**95% CI**	
					**Lower**	**Upper**
Diabetes mellitus	-1.058	0.322	0.001	0.347	0.185	0.652
Antiplatelets	-0.111	0.479	0.817	0.895	0.350	2.286
NLR	-0.036	0.027	0.173	0.964	0.915	1.016
"A" antigen	1.494	0.301	<0.001	4.454	2.467	8.040
"B" antigen	1.674	0.318	<0.001	5.333	2.857	9.954
Inferior/anterior wall MI	-1.615	1.199	0.178	0.199	0.019	2.087
Lateral /anterior wall MI	-1.007	1.206	0.404	0.365	0.034	3.884
Constant	1.044	1.188	0.380	2.840		
**Outcome Variable: MACCE**	**Coefficients**	**SE**	***P*** **value**	**Odds Ratio**	**95% CI**	
					**Lower**	**Upper**
Diabetes mellitus	1.182	0.396	0.003	3.262	1.502	7.083
Antiplatelets	0.616	0.563	0.274	1.852	0.615	5.578
NLR	-0.021	0.044	0.632	0.979	0.898	1.068
"A" antigen	-0.357	0.406	0.379	0.699	0.316	1.550
"B" antigen	-0.419	0.429	0.329	0.658	0.284	1.524
Constant	-21.011	17849	0.999	<0.001		

## Discussion

This study assessed the association between ABO antigen and ST-segment recovery among patients with STEMI treated with fibrinolysis. Although the prediction of ST-segment resolution following thrombolysis has been a challenge to the cardiologists, it has been believed that complete ST-segment resolution in ECG after thrombolysis generally improves the prognosis of STEMI.^3,13^ The main and interesting finding of our study is that O blood group is associated with poor reperfusion response in patients with acute STEMI treated with thrombolysis as evident by less ST-segment recovery.

Several previous studies revealed the relation between ABO blood groups and risk of CAD.^6-8,17,18^ In accordance with our findings, Biswas et al established type-O blood as a risk factor for CADs in Bangladeshi people [OR: 2.034 (1.127-3.67)].^19^ Furthermore, Ketch et al in a cohort study on 1198 patients who underwent PCI for acute myocardial infarction, showed higher prevalence of atherosclerosis and prior history of PCI among patients with O blood type, despite of no difference in procedural success, in-hospital blood transfusion, and incident MACE after 1 year follow-up.^20^ On the other hand, in a study by Carpeggiani et al in Italy, it has been reported that non-O blood group is associated with increased risk for cardiac death and mortality.^7^ Zhou et al in another study among 2708 patients, suggested that A blood group was an independent risk factor for the presence and severity of CAD.^21^ According to the findings of a meta-analysis study, non-O blood group increased risk of myocardial infarction by 25% (95% CI: 1.14–1.36); however, by focusing only on the results of prospective studies, this association was not confirmed (OR 1.01; 95% CI: 0.84–1.23).^22^ Recently, Lin et al demonstrated that blood type O is associated with spontaneous reperfusion of the occluded coronary artery in acute myocardial infarction patients^23^; which was in contrast to our findings. However, Askin et al reported no association between the ABO blood type and thrombus burden in STEMI patients.^24^ Another study in the Netherlands did not find any correlation between ABO blood groups and cardiovascular complications or long-term mortality during four years of follow-up after vascular surgery.^25^ Likewise, Ketch et al showed that the rate of recurrent thrombotic events including myocardial infarction, stent thrombosis, and target vessel revascularization was similar in patients with non-O and O blood types.^20^ It has been well accepted that the distribution of ABO blood groups are distinct in different ethnic population.^26^ Thus, the possible reason for these discrepancies might be due to the diversity of population and ethnic differences of these studies.

Regarding dyslipidemia as an independent risk factor for incident IHD,^27^ ABO blood groups are suggested to associate with atherosclerotic cardiovascular diseases, through the effects of soluble intercellular adhesion molecule-1 (sICAM-1), soluble P-selectin (sP-selectin), and soluble E-selectin (sE-selectin).^28,29^ Many studies reported the association of ABO antigens with serum cholesterol and low-density lipoprotein cholesterol levels.^30,31^ Ketch et al also reported the higher prevalence of hypercholesterolemia among those with O blood type.^20^ Taken together, poor response to thrombolytic therapy in patients with O blood type may be attributable to the higher burden of atherosclerosis and hypercholesterolemia in these patients, which may lead to a less favorable response to fibrinolysis. Of course, in the present study angiographic assessment of coronary artery involvement was not performed, so this possible mechanism is only hypothesis generating and needs detailed coronary angiographic data.

The mechanisms through how ABO antigens may participate in the pathogenesis of CAD and myocardial infarction remain unproved. Most of the familial CAD might be linked to heritable risk factors, and the inheritance of ABO antigens could have important roles in this condition. The effects of blood group antigens on the level of inflammatory proteins and their central role for inflammation in all phases of the atherosclerotic process have been previously identified.^32,33^ It is well-known that inflammation may increase the presence and progression of cardiovascular diseases, probably through mediating C-reactive protein, interleukin-6 and tumor necrosis factors.^34^

Moreover, the etiology of an acute coronary syndrome (ACS) varies from embolization of a fractured atheroma to a sudden occlusion of the coronary artery by a fresh clot on the thrombogenic surface of chronic atheromatous lesion.^35^ The less frequent causes of ACS may include spasm of coronary arteries and embolization of calcium or vegetations. The response to thrombolytic therapy depends on the nature of the occluding lesion and burden of thrombus. Lesions with higher thrombus burden are more likely to respond to the administration of thrombolysis. The presence of A or B antigens in peripheral blood are considered a risk factor to hypercoagulability. Topcu et al investigated thrombus burden in patients with STEMI undergoing primary PCI.^36^ Non-O blood group associated with high angiographic thrombus burden that may explain the higher response rate to thrombolysis among patients with Blood groups of A, B and AB. On the other hand in patients with O blood group the presence of smaller thrombus burden as the main occluding lesion correlates with a less favorable response to thrombolysis in patients with this blood group.

On the other hand, Von Will brand factor (vWF) plays an important role in hemostasis and thrombosis by mediating platelet adhesion to the vascular wall, especially under high shear stress action.^37^ It has been shown that this factor participates in platelet aggregation along with fibrinogen^37^; thus, the observed increased risk of non-O blood group for CAD in the literature could be attributable to higher vWF levels in these patients, which has been reported to be approximately 25% higher in non-O vs. O-blood groups in several studies.^20,38^ Furthermore, a relationship between Factor VIII (FVIII) plasma concentrations and ABO blood groups has been seen, which is suggested to be mediated via VWF.^39^

Some limitations may exist in the current study. First, the study is subject to the limitations inherent to its cross-sectional, small sample size and single center non-randomized design. Second, we exclude patients who underwent primary angioplasty; thus, angiographic measures like number of involved vessels, coronary thrombolysis in myocardial infarction flow and myocardial blush grade remains unknown in the present study, and we relied on ST resolution as the only surrogate for myocardial reperfusion after thrombolysis. In addition, we did not measure vWF level, which may have prognostic value independent of ABO blood groups. So, larger well-powered studies are needed to confirm the results of the present study.

## Conclusion

We concluded that the presence of A or B blood group antigens is associated with a better response to thrombolytic therapy in patients with acute STEMI. This finding may imply a higher thrombotic component of coronary artery occlusion in these patients.

## Competing interests

No conflict of interest has been declared by the authors.

## Ethical approval

The study design, protocols, and informed consent forms were reviewed and approved by the institutional review board for its merit and ethics in human subject research. All patients signed a partial waiver form for Health Insurance Portability and Accountability Act to allow the investigating team to screen their medical records for study eligibility. Following the screening process, all patients who met the inclusion criteria were asked to sign consent for the study prior to initiation of thrombolysis.

## Funding

No funding has been requested for this study.
